# A Review of First Line Infertility Treatments and Supporting Evidence in Women with Polycystic Ovary Syndrome

**DOI:** 10.3390/medsci7090095

**Published:** 2019-09-10

**Authors:** Michael Costello, Rhonda Garad, Roger Hart, Hayden Homer, Louise Johnson, Cailin Jordan, Edgar Mocanu, Jie Qiao, Luk Rombauts, Helena J. Teede, Eszter Vanky, Christos Venetis, William Ledger

**Affiliations:** 1 University of New South Wales, High St, Kensington, Sydney, NSW 2033, Australia; 2 Monash Centre for Health Research and Implementation, Monash Public Health and Preventive Medicine, Monash University, and Monash Health, Melbourne, VIC 3168, Australia; 3 Division of Obstetrics and Gynaecology, The University of Western Australia, University of Western Australia, Crawley, WA 6008, UK; 4 Christopher Chen Oocyte Biology Research Laboratory, UQ Centre for Clinical Research, The University of Queensland, Brisbane, QLD 4029, Australia; 5 Victorian Assisted Reproductive Treatment Authority, Melbourne, VIC 3000, Australia; 6 Genea Hollywood Fertility, Wembley 6014, Australia; 7 Royal College of Surgeons, Rotunda Hospital, Dublin D02 YN77, Ireland; 8 Department of Obstetrics and Gynaecology, Medical Center for Human Reproduction, Peking University Third Hospital, Beijing 100191, China; 9 Department of Obstetrics and Gynaecology, Monash University, Melbourne 3168, Australia; 10 National Health and Medical Research Council Centre for Research Excellence in PCOS, Monash University, Melbourne, VIC 3168, Australia; 11 Department of Clinical and Molecular Medicine, Norwegian University of Science and Technology, 1517 Trondheim, Norway

**Keywords:** polycystic ovary syndrome, infertility, treatment, ovulation induction

## Abstract

Polycystic ovary syndrome (PCOS) is the most common cause of anovulatory infertility in women of reproductive age. Lifestyle change is considered the first line treatment for the management of infertile anovulatory women with PCOS, and weight loss for those who are overweight or obese. First line medical ovulation induction therapy to improve fertility outcomes is letrozole, whilst other less efficacious ovulation induction agents, such as clomiphene citrate, metformin, and metformin combined with clomiphene citrate, may also be considered. Metformin combined with clomiphene citrate is more effective than clomiphene citrate alone. In obese women with PCOS, clomiphene citrate could be used in preference to metformin alone whilst clomiphene citrate could be added to metformin alone in order to improve reproductive outcome in all women with PCOS. Gonadotrophins, which are more effective than clomiphene citrate in therapy naïve women with PCOS, can be considered a first line therapy in the presence of ultrasound monitoring, following counselling on the cost and the potential risk of multiple pregnancy.

## 1. Introduction

Polycystic ovary syndrome (PCOS), with a prevalence of between 8% and 13%, depending on the population studied and definitions used, is the most common endocrinopathy affecting women of reproductive age. PCOS is a complex condition with reproductive, metabolic, and psychological features [1]. A diagnosis of PCOS is based on features of anovulation and/or oligo-ovulation, clinical or biochemical hyperandrogenism, and polycystic ovaries [2]. 

Infertility is a prevalent presenting feature of PCOS with 75% of these women experiencing infertility due to anovulation, making PCOS the most common cause of anovulatory infertility [3]. The treatment of infertility in PCOS includes lifestyle changes (diet, exercise, and behavioural strategies), pharmacological therapies (oral agents such as clomiphene citrate, letrozole or metformin or injectable agents, such as gonadotrophins), surgical therapy (laparoscopic ovarian surgery) or in vitro fertilization (IVF) [4]. In vitro maturation (IVM) has been proposed to offer a promising alternative to conventional IVF [5].

The aim of this narrative review, is to provide a summary, and brief update of, the best available and most current research evidence evaluating the first line treatment of PCOS related infertility, and that which informed the recommendations in the assessment and treatment of infertility section of the International Evidence-based Guideline on PCOS [6,7,8,9]. The methodology for the literature search and selection of studies for this narrative review are described in the guidelines and the guidelines’ technical report. The Grading of Recommendations, Assessment, Development, and Evaluation (GRADE) framework was applied across the quality of evidence (QOE) [10]. 

## 2. Lifestyle Treatment

### 2.1. Background

Lifestyle interventions (including diet, exercise, and behavioural management techniques) which reduce weight by as little as 5% of total body weight have been shown to have health, metabolic, reproductive, and psychological benefits in women with PCOS and excess weight [9].

### 2.2. Evidence

A Cochrane systematic review published in 2011, comparing lifestyle interventions (diet, exercise, and behavioural management techniques for modifying diet or exercise, or a combination of these) versus minimal intervention in women with PCOS, located six randomized controlled trials (RCTs), of which no studies reported on live birth, two studies reported on pregnancy data, and three studies reported on ovulation [11]. Of the two RCTs reporting on pregnancy, only two of 20 women and none of 51 women were actively seeking pregnancy, making any comparison of pregnancy rates between the two interventions meaningless. Three RCTs reported on ovulation in overweight or obese PCOS women with none showing a significant improvement in ovulation rate, and unfortunately the data was in a form inappropriate for meta-analysis.

### 2.3. Summary

Despite limited supporting level 1 (RCTs or systematic reviews of RCTs) evidence, lifestyle intervention is considered the first line treatment for the management of infertile anovulatory women with PCOS, and weight loss for those who are overweight or obese, due to its health, metabolic, reproductive and psychological benefits.

## 3. Clomiphene Citrate

### 3.1. Background 

Clomiphene citrate, a selective estrogen receptor modulator, was first reported to induce ovulation in 1961 [12] and has been used as a first-line medical ovulation induction agent since 1967 [13]. Clomiphene citrate is administered for 5 days, beginning on any menstrual cycle day from two to five, starting with 50 mg/day and increasing to 150 mg/day if anovulatory. If ovulation cannot be achieved at doses of 150 mg/day, the patient is deemed to have clomiphene citrate resistance. If pregnancy cannot be achieved after six ovulatory cycles, then the patient is deemed to have clomiphene citrate failure [14]. Studies evaluating clomiphene citrate have shown an ovulation rate of 60% to 85% and a pregnancy rate of 30% to 50% after six ovulatory cycles, with an increased risk of multiple pregnancy (5% to 7%) [15]. 

### 3.2. Evidence

#### Clomiphene Citrate Versus Placebo/No Treatment

A Cochrane systematic review and pairwise meta-analysis in women with WHO Group 2 anovulation comparing clomiphene citrate with placebo demonstrated that clomiphene citrate improves the clinical pregnancy rate per woman (OR 5.91; 95% CI 1.77–19.68; *p* = 0.004; three RCTs; 133 women, low QOE) [16]. The first ever systematic review and network meta-analysis of RCTs on ovulation induction also compared clomiphene citrate versus placebo/no treatment and found a higher pregnancy (OR 0.30; 95% CI 0.15-0.58) and ovulation (OR 0.14; 95% CI 0.07–0.30) rate with clomiphene citrate in WHO Group 2 (including PCOS) anovulatory women [17].

### 3.3. Summary

Clomiphene citrate could be used alone as first line pharmacological therapy in anovulatory women with PCOS and no other infertility factors, as it is superior to placebo or no treatment.

## 4. Metformin

### 4.1. Background

Metformin, an insulin sensitizing drug, was first reported as a treatment for PCOS in 1994, where it was found to facilitate normal menses and pregnancy [18]. The first systematic review to report on metformin’s use in PCOS demonstrated, based on limited observational studies and RCTs on predominately obese women with PCOS, that metformin alone improved both restoration of regular menses and spontaneous ovulation, but there was no data supporting an improvement in pregnancy rate [19].

### 4.2. Evidence

#### 4.2.1. Metformin Versus Placebo/No Treatment

A recent Cochrane systematic review and pairwise meta-analysis of RCTs comparing metformin with placebo or no treatment in PCOS women showed that metformin improves live birth (OR 1.59; 95% CI 1.00–2.51; *p* = 0.049; four RCTs; 435 women; low QOE), clinical pregnancy (OR 1.93; 95% CI 1.42–2.64; nine RCTs; 1027 women; moderate QOE), and ovulation (OR 2.55; 95% CI 1.81–3.59; 14 RCTs; 701 women; moderate QOE; I^2^ = 58%) rates per woman with no evidence of a difference in the miscarriage rate per woman (OR 1.08; 95% CI 0.50–2.35; four RCTs; 748 women; low QOE), but there was an increased risk of adverse events (gastrointestinal side-effects) per woman with metformin (OR 4.76; 95% CI 3.06–7.41; seven RCTs; 670 women; moderate QOE; I^2^ = 61%) [20].

#### 4.2.2. Metformin Versus Other Ovulation Induction Therapies

Metformin has been compared in a head to head manner to clomiphene citrate in the most recent Cochrane systematic review and pair-wise meta-analyses of RCTs [20]. In obese (BMI ≥ 30 kg/m^2^) women with PCOS, clomiphene citrate is superior to metformin for the outcomes of live birth (OR 0.30; 95% CI 0.17–0.52; two RCTs; 500 women: very low QOE), clinical pregnancy (OR 0.34; 95% CI 0.21-0.55; two RCTs; 500 women: very low QOE), and ovulation (OR 0.29; 95% CI 0.20–0.43; two RCTs; 500 women: low QOE) rates per woman, with insufficient evidence for a difference in multiple pregnancy (OR 0.14; 95% CI 0.01–2.76; two RCTs; 500 women: very low QOE) or miscarriage (OR 0.61; 95% CI 0.27–1.38; two RCTs; 500 women: low QOE) rates per woman.

This most recent Cochrane systematic review also compared metformin versus clomiphene citrate in non-obese (BMI < 30 kg/m^2^) women with PCOS and found an increased clinical pregnancy rate per woman randomized to metformin (OR 1.56; 95% CI 1.05–2.33; five RCTs; 490 women; very low QOE) but no evidence of a difference in live birth (OR 1.71 favouring metformin; 95% CI 1.00–2.94; *p* = 0.052; three RCTs; 241 women; I^2^ 78%; very low QOE), ovulation (OR 0.81; 95% CI 0.51–1.28; four RCTs; 312 women), miscarriage (OR 1.58; 95% CI 0.61–4.09; three RCTs; 241 women) and multiple pregnancy (OR 0.46; 95% CI 0.07–3.16; 3 RCTs; 358 women) per woman randomized [20].

### 4.3. Summary

Metformin could be used alone as first line pharmacological therapy in anovulatory women with PCOS and no other infertility factors, as it is superior to placebo or no treatment, although it is less effective than clomiphene citrate in obese PCOS women.

## 5. Metformin Combined with Clomiphene Citrate

### 5.1. Evidence

#### 5.1.1. Metformin Combined with Clomiphene Citrate Versus Placebo/No Treatment

Both the recently published international evidence based guidelines on PCOS [9] and the recently published systematic review and network meta-analysis of RCTs on ovulation induction in WHO Group 2 (including PCOS) anovulatory women [17], did not identify any published RCTs directly comparing metformin combined with clomiphene citrate versus placebo/no treatment. However, the latter systematic review demonstrated a higher pregnancy (OR 6.11; 95% CI 3.02–12.38) and ovulation (OR 10.91; 95% CI 4.86–24.48) rate with metformin combined with clomiphene citrate, but insufficient evidence to determine whether there was a difference in live birth (OR 4.66; 95% CI 0.59–36.68), miscarriage (OR 1.53; 95% CI 0.32–7.28) or multiple pregnancy (OR 0.85; 95% CI 0.02–30.70) rates per woman between the two interventions upon network meta-analysis. This network meta-analysis also showed a higher pregnancy rate (OR 5.86; 95% CI 1.17–29.29) per randomized woman in therapy naïve WHO Group 2 (including PCOS) anovulatory women.

#### 5.1.2. Metformin Combined with Clomiphene Citrate Versus Other Ovulation Induction Therapies

Two recently published systematic reviews conducting pairwise meta-analyses of RCTs have compared metformin combined with clomiphene citrate versus clomiphene citrate alone in women with PCOS [20] and WHO Group 2 (including PCOS) anovulatory women [17]. Both Morley et al and Wang et al demonstrated an improvement in pregnancy (Morley et al: clinical pregnancy: OR 1.59; 95% CI 1.27–1.99; 16 RCTs; 1529 women; moderate QOE and Wang et al pregnancy: OR 1.56; 95% CI 1.24–1.97; 19 RCTs; 2070 women) and ovulation (OR 1.57; 95% CI 1.28–1.92; 21 RCTs; 1624 women; I^2^ 64%; moderate QOE and OR 1.46; 95% CI 1.01–2.12; 14 RCTs; 1407 women; I^2^ 54.5%, respectively) rates per woman randomized to metformin combined with clomiphene citrate, but there was no evidence of a difference in live birth (OR 1.21 favouring metformin combined with clomiphene citrate; 95% CI 0.92–1.59; nine RCTs; 1079 women; low QOE and OR 1.14 favouring metformin combined with clomiphene citrate; 95% CI 0.81–1.61; seven RCTs; 950 women, respectively) or multiple pregnancy (OR 0.56; 95% CI 0.18-1.68; six RCTs; 1003 women and OR 0.57; 95% CI 0.19–1.74; four RCTs; 892 women, respectively) rates per woman randomized between the two treatments. However, Morley et al found a higher miscarriage rate per woman randomized (OR 1.59; 95% CI 1.03–2.46; nine RCTs, 1096 women; low QOE) with metformin combined with clomiphene citrate, whilst Wang et al found insufficient evidence for a difference in miscarriage rate per woman randomized between the two therapies (OR 1.38; 95% CI 0.85–2.24; eight RCTs; 991 women). Wang and colleagues also performed a network meta-analysis of RCTs comparing metformin combined with clomiphene citrate versus clomiphene citrate alone in WHO Group 2 (including PCOS) anovulatory women and found a higher pregnancy (OR 1.81; 95% CI 1.35–2.42) and ovulation (OR 1.55; 95% CI 1.02–2.36) rate per woman with metformin combined with clomiphene citrate, but insufficient evidence to suggest a difference in live birth (OR 1.45; 95% CI 0.88–2.41), miscarriage (OR 1.16; 95% CI 0.63–2.16) or multiple pregnancy (OR 0.57; 95% CI 0.20–1.59) rates between the two treatments.

If metformin alone is being used for ovulation induction in women with PCOS who are obese (BMI ≥ 30 kg/m^2^), the addition of clomiphene citrate improves live birth (OR 0.23; 95% CI 0.13–0.40; two RCTs; 741 women: low QOE), pregnancy (OR 0.23; 95% CI 0.14-0.37; two RCTs; 741 women: low QOE) and ovulation (OR 0.23; 95% CI 0.15–0.34; two RCTs; 741 women: low QOE) rates with no evidence of a difference in miscarriage ( OR 0.47; 95% CI 0.22–1.00; two RCTs; 741 women: low QOE) or adverse events (OR 0.86; 95% CI 0.23-3.04; two RCTs; 741 women: low QOE) rates per woman [14].

### 5.2. Summary

Metformin combined with clomiphene citrate could be used as first line pharmacological therapy in anovulatory women with PCOS and no other infertility factors, as it is superior to placebo or no treatment and also more effective than clomiphene citrate alone and metformin alone (obese women). 

## 6. Letrozole

### 6.1. Background

Letrozole, an aromatase inhibitor, was first proposed as an ovulation inducing drug in anovulatory PCOS women in 2001 Table S1 [21]. Letrozole is usually administered on days 3 to 7 of the menstrual cycle at doses of 2.5 to 7.5 mg per day in 2.5 mg increments [13]. Initially, there were concerns regarding the potential teratogenic effect of letrozole for infertility treatment [22] but a recent review of the published literature did not find an increased risk of foetal anomalies with letrozole [17].

### 6.2. Evidence

#### 6.2.1. Letrozole Versus Placebo or No Treatment

The recently published international evidence based guideline on PCOS identified a single published RCT comparing letrozole versus placebo or no treatment with timed intercourse in PCOS [23]. This RCT showed insufficient evidence for a difference between letrozole and placebo in live birth (1/18 women; 6% versus 0/18 women; 0% respectively; *p* = 0.324) and clinical pregnancy (1/18 women; 6% versus 0/18 women; 0% respectively; *p* = 0.324) rates but a higher ovulation rate (6/18 women; 33% versus 0/18 women; 0% respectively; *p* = 0.006) per woman with letrozole in clomiphene citrate resistant PCOS women. There were no cases of miscarriage or multiple pregnancy in the RCT.

In the recently published systematic review and network meta-analysis on ovulation induction, letrozole was shown to be more effective than placebo or no treatment in terms of pregnancy (OR 5.35; 95% CI 2.63–10.87) and ovulation (OR 13.99; 95% CI 6.23–31.42) rates with insufficient evidence to suggest a difference in live birth (OR 5.34 in favour of letrozole; 95% CI 0.67–42.30), miscarriage (OR 1.33; 95% CI 0.26–6.91) and multiple pregnancy (OR 0.69; 95% CI 0.02–25.09) rates per randomized woman for WHO Group 2 (including PCOS) anovulatory women. Letrozole was also superior to placebo or no treatment for the primary outcome of pregnancy rate (OR 6.39; 95% CI 1.29–31.73) in therapy naïve WHO Group 2 (including PCOS) anovulatory women [17].

#### 6.2.2. Letrozole Versus Metformin

In the systematic review and network meta-analysis on ovulation induction, letrozole was shown to be more effective than metformin in terms of live birth rate (OR 0.54; 95% CI 0.29–0.98) for WHO Group 2 (including PCOS) anovulatory women [17].

#### 6.2.3. Letrozole Versus Clomiphene Citrate

In this systematic review and network meta-analysis on ovulation induction, letrozole was also shown to be superior to clomiphene citrate in terms of live birth (OR 1.67; 95% CI 1.11–2.49), pregnancy (OR 1.58; 95% CI 1.25–2.00) and ovulation (OR 1.99; 95% CI 1.38–2.87) rates for WHO Group 2 (including PCOS) anovulatory women and for pregnancy rate (OR 1.80; 95% CI 1.20–2.70) in therapy naïve WHO Group 2 (including PCOS) anovulatory women [17]. Current evidence supports no difference in miscarriage rates between letrozole and clomiphene citrate, albeit with wide 95% confidence intervals (OR 1.01; 95% CI 0.58–1.75). A pairwise meta-analysis was also performed in this systematic review and demonstrated a higher live birth (OR 1.60; 95% CI 1.30–1.98; nine RCTs; 1990 women), pregnancy (OR 1.53; 95% CI 1.26–1.85; 21 RCTs; 3553 women) and ovulation (OR 1.89; 95% CI 1.55–2.30; 14 RCTs; 2568 women) rates per woman with letrozole compared to clomiphene citrate but no difference in miscarriage rates, albeit with wide 95% confidence intervals (OR 1.00; 95% CI 0.62–1.62; 10 RCTs; 2302 women). In addition, the risk of multiple pregnancy per woman randomized was lower with letrozole compared to clomiphene citrate (network meta-analysis result: OR 0.46; 95% CI 0.23–0.92; pairwise meta-analysis result: OR 0.45; 95% CI 0.22–0.91; 12 RCTs; 2460 women) in WHO Group 2 (including PCOS) women.

This network meta-analysis, which compared seven ovulation induction therapies plus placebo or no treatment, demonstrated that (i) letrozole was superior to clomiphene citrate for all the important outcomes of ovulation, pregnancy, live birth and multiple pregnancy rates in WHO Group 2 (including PCOS) anovulatory women; (ii) letrozole was the only ovulation induction therapy superior to clomiphene citrate in terms of live birth rate for WHO Group 2 (including PCOS) anovulatory women and letrozole was the only ovulation induction treatment superior to clomiphene citrate in (iii) therapy naïve WHO Group 2 (including PCOS) anovulatory women and (iv) high quality RCTs with low risks of both randomization and allocation concealment bias [17].

A more recently published Cochrane systematic review and pairwise meta-analysis of RCTs comparing letrozole versus clomiphene citrate in PCOS women [24] also demonstrated a significantly higher live birth (OR 1.79; 95% CI 1.42–2.25; eight RCTs; 1646 women; low QOE) and clinical pregnancy (OR 1.50; 95% CI 1.28–1.76; 17 RCTs; 2930 women; low QOE) rate per woman randomized with letrozole with insufficient evidence to determine if there was a difference in multiple pregnancy (OR 0.61 favouring letrozole; 95% CI 0.32-1.16; 13 RCTs; 2409 women; moderate QOE) or miscarriage (OR 1.37 favouring clomiphene citrate; 95% CI 0.97-1.93; 11 RCTs; 2190 women; moderate QOE) rates per woman randomized.

Another recently published systematic review and pairwise meta-analysis of RCTs comparing letrozole versus clomiphene citrate in PCOS women demonstrated similar findings to the above Cochrane Review [25]. This review found a significantly higher live birth (RR 1.55; 95% CI 1.28–1.88; six RCTs; 1400 women) and pregnancy (RR 1.34; 95% CI 1.09–1.64; nine RCTs; 2045 women) rate per woman randomized with letrozole with insufficient evidence to determine if there was a difference in multiple pregnancy (RR 0.43 favouring letrozole; 95% CI 0.17–1.07; five RCTs; 1598 women) or miscarriage (RR 1.36 favouring clomiphene citrate; 95% CI 0.98–1.89; eight RCTs; 1939 women) rates per woman randomized.

#### 6.2.4. Letrozole Versus Clomiphene Citrate Combined with Metformin

The Cochrane systematic review and pairwise meta-analysis of RCTs also compared letrozole to clomiphene citrate combined with metformin [24] and identified a single RCT of clomiphene citrate resistant women with PCOS [26], and therefore, the findings of that RCT are not relevant to this paper’s scope of reviewing first line therapies for anovulatory women with PCOS.

#### 6.2.5. Letrozole Versus Letrozole Combined with Clomiphene Citrate

A single recently published pilot RCT, powered for the primary outcome measure of ovulation rate, compared letrozole with letrozole combined with clomiphene citrate over one treatment cycle in 70 infertile PCOS women with a mean BMI of 33–34 kg/m^2^ of whom 81% had been treated with letrozole or clomiphene citrate in the past and 40% were currently taking metformin during the trial [27]. The results showed a higher ovulation rate with letrozole combined with clomiphene citrate compared to letrozole alone (77.1% versus 42.9%; RR 1.80; 95% CI 1.18–2.75; *p* = 0.007), but there was insufficient evidence for a difference in clinical pregnancy (2.9% versus 9.1%; RR 3.09; 95% CI 0.34– 28.23; *p* = 0.356) or live birth (7% versus 12%; RR 1.68; 95% CI 0.19–14.66; *p* = 1.00) rates between the two interventions. There were no multiple pregnancies for either therapy.

### 6.3. Summary

Letrozole should be considered the first line pharmacological treatment of choice for ovulation induction in anovulatory women with PCOS and no other infertility factors, as it is superior to placebo or no treatment, metformin, clomiphene citrate and clomiphene citrate combined with metformin. Large, adequately powered, well conducted and reported RCTs are required, to compare letrozole combined with metformin or clomiphene citrate to letrozole alone in anovulatory women with PCOS, given that there are no published RCTs comparing these interventions with respect to metformin, and that there is only a single published RCT powered for ovulation only with respect to clomiphene citrate.

## 7. Gonadotrophins

### 7.1. Background

Ovulation induction with gonadotrophins began in the 1960s and there is a large body of observational evidence supporting the use of gonadotrophin ovulation induction in clomiphene citrate resistant or clomiphene citrate failure women with PCOS [28].

### 7.2. Evidence

#### 7.2.1. Gonadotrophins Versus Placebo/No Treatment

The recently published international evidence based guideline on PCOS [9] and the recently published systematic review and network meta-analysis of RCTs on ovulation induction in WHO Group 2 (including PCOS) anovulatory women [17] did not find any published RCTs directly comparing gonadotrophins versus placebo/no treatment. However, the latter systematic review and network meta-analysis showed a higher pregnancy rate (OR 6.08; 95% CI 1.05–35.25) per randomized woman with gonadotrophins compared to placebo/no treatment in therapy naïve WHO Group 2 (including PCOS) anovulatory women.

#### 7.2.2. Gonadotrophins Versus Other Ovulation Induction Therapies

A Cochrane systematic review and pairwise meta-analysis in therapy naïve PCOS anovulatory women comparing gonadotrophins to clomiphene citrate demonstrated that gonadotrophins are superior in terms of both live birth/ongoing pregnancy(OR 0.64; 95% CI 0.41–0.98; two RCTs; 378 women) and clinical pregnancy (OR 0.61; 95% CI 0.40–0.93; two RCTs; 378 women) rates per woman with inconclusive results showing no evidence of a difference in miscarriage (OR 0.84; 95% CI 0.39–1.78; three RCTs; 696 women), multiple pregnancy (OR 0.26 favouring clomiphene citrate; 95% CI 0.06–1.06; three RCTs; 696 women), and ovarian hyperstimulation syndrome (OR 0.19; 95% CI 0.02–1.67; two RCTs; 396 women) rates per woman [16].

The recently published international evidence based guideline on PCOS [9] and the recently published systematic review and network meta-analysis of RCTs on ovulation induction [17] did not find any published RCTs directly comparing gonadotrophins versus clomiphene citrate combined with metformin in therapy naïve PCOS women and therapy naïve WHO Group 2 (including PCOS) anovulatory women, respectively. The network meta-analysis showed no evidence of a difference between these two treatments in the primary outcome measure of pregnancy rate (OR 1.04; 95% CI 0.40–2.67) per randomized woman in therapy naïve WHO Group 2 (including PCOS) anovulatory women, although the results were inconclusive due to wide 95% confidence intervals [17].

A network meta-analysis comparing letrozole versus gonadotrophins also found no evidence of a difference in reproductive outcomes, including the primary outcome of pregnancy rate per randomized woman for therapy naïve WHO Group 2 (including PCOS) anovulatory women (OR 0.95; 95% CI 0.39–2.31), with the results being inconclusive due to the wide 95% confidence intervals [17].

### 7.3. Summary

Gonadotrophins could be considered as first line pharmacological therapy in anovulatory women with PCOS and no other infertility factors, as it is superior to both placebo or no treatment and clomiphene citrate in therapy naïve women, in the presence of ultrasound monitoring, following counselling on cost and the potential risk of multiple pregnancy. Large, adequately powered, well conducted and reported RCTs comparing both gonadotrophins against combined clomiphene citrate and metformin and gonadotrophins against letrozole in therapy naïve anovulatory women with PCOS are needed.

## 8. Conclusions

The evidence and summaries for first line ovulation induction in this review are applicable to women with PCOS who have anovulatory infertility and no other infertility factors. Lifestyle intervention is considered the first line treatment if women are overweight or obese. Letrozole should be considered the first line pharmacological treatment for ovulation induction. However, other less efficacious ovulation induction agents, such as clomiphene citrate, metformin, and metformin combined with clomiphene citrate may also be considered, as they are superior to placebo or no treatment. Metformin is less effective than clomiphene citrate in obese women with PCOS. Metformin combined with clomiphene citrate is more effective than clomiphene citrate alone and metformin alone (obese women). Gonadotrophins, which are more effective than clomiphene citrate in therapy naïve women with PCOS, can be considered as a first line therapy in the presence of ultrasound monitoring, following counselling on cost and potential risk of multiple pregnancy. Research priorities in the form of large, adequately powered, well conducted and reported RCTs are required to evaluate optimal lifestyle interventions, and also those comparing letrozole combined with metformin or clomiphene citrate against letrozole alone and gonadotrophins against letrozole in therapy naïve anovulatory infertile women with PCOS. 

## Supplementary Materials

The following are available online at https://www.mdpi.com/2076-3271/7/9/95/s1, Table S1: First Line Treatments of Anovulatory Infertility in Polycystic Ovary Syndrome.
