# Supplementary material for: A Review of First Line Infertility Treatments and Supporting Evidence in Women with Polycystic Ovary Syndrome
Source: Med Sci (Basel). 2019 Sep 10;7(9):95. doi: 10.3390/medsci7090095 (PMC6780967; doi:10.3390/medsci7090095)
Supplement: Supplementary file 1 [file medsci-07-00095-s001.pdf]

Supplementary Material

Review

A Review of First Line Infertility Treatments and Supporting Evidence in Women with Polycystic Ovary Syndrome

Table S1: First Line Treatments of Anovulatory Infertility in Polycystic Ovary Syndrome.

**LIFESTYLE**
• **Lifestyle intervention (including diet, exercise and behavioural management techniques)** is first line treatment with weight loss for those women with excess weight, due to its health, metabolic, reproductive and psychological benefits.
**PHARMACOLOGICAL OVULATION INDUCTION THERAPY**
• **Letrozole** is first line. Cumulative live birth rates over 5 treatment cycles have been reported for letrozole (27.5%) (see footnote below) [1].<br>• Other less effective therapies may also be considered including **clomiphene citrate**, **metformin**, or **metformin combined with clomiphene citrate.** Cumulative live birth rates over 6 treatment cycles have been reported for metformin (7.2%), clomiphene citrate (22.5%) and metformin combined with clomiphene citrate (26.8%) respectively (see footnote below) [2].<br>1. **metformin combined with clomiphene citrate** is more effective than **clomiphene citrate** and **metformin** (obese women).<br>2. **clomiphene citrate** is more effective than metformin (obese women)<br>• **Gonadotrophins** could also be considered as first line, as it is more effective than clomiphene citrate, in the presence of ultrasound monitoring, following counselling on cost and potential risk of multiple pregnancy. Cumulative live birth /ongoing pregnancy rates over 3 treatment cycles have been reported for clomiphene citrate (29.8%) and Gonadotrophins (40%) respectively (see footnote below) [3].

Footnote: The below RCTs or systematic review of RCTs on infertile women with PCOS reported on the important outcomes of cumulative live birth or ongoing pregnancy rates. It is not valid to compare the outcomes

between these studies as such comparisons are indirect due to differences in population, interventions, comparisons and outcomes between the different studies.

1. The RCT by Legro et al 2014 compared clomiphene citrate versus letrozole in women with PCOS (mean age of 29 years and mean BMI of 35 kg/m^2) [1].

2. The RCT by Legro et al 2007 compared metformin versus clomiphene citrate versus metformin combined with clomiphene citrate in women with PCOS (mean age of 28 years and mean BMI of 35 kg/m^2) [2].

3. The Systematic Review and meta-analysis of RCTs by Brown et al 2016 compared clomiphene citrate versus Gonadotrophins in therapy naïve women with PCOS (mean age of 30 years and mean BMI of 23.5 kg/m^2) [3].

References

1. Legro, R.S.; Brzyski, R.G.; Diamond, M.P.; Coutifaris, C.; Schlaff, W.D.; Casson, P., Christman, G.M., Huang, H., Yan, Q., Alvero, R.; et al. Letrozole versus clomiphene for infertility in the polycystic ovary syndrome. *N. Engl. J. Med.* **2014**, *371*, 119.
2. Legro RS, Barnhart HX, Schlaff WD, Carr BR, Diamond MP, Carson SA, et al. Clomiphene, Metformin, or Both for Infertility in the Polycystic Ovary Syndrome. *N. Engl. J. Med.* **2007**, *356*, 551–566.
3. Brown, J.; Farquhar, C. Clomiphene and other antioestrogens for ovulation induction in polycystic ovarian syndrome. *Cochrane Database Syst. Rev.* **2016**, *12*, CD002249.
